# msuav500k: Foundational dataset for multispectral and RGB uncrewed aerial vehicle imagery

**DOI:** 10.1016/j.dib.2025.112128

**Published:** 2025-10-09

**Authors:** Jurrian Doornbos, Önder Babur

**Affiliations:** a Wageningen University and Research, Information Technology, Hollandseweg 1, 6706 KN Wageningen, The Netherlands; b Eindhoven University of Technology, Department of Mathematics and Computer Science, MetaForum, De Groene Loper 5, 5612 AZ Eindhoven, The Netherlands

**Keywords:** Multispectral imagery, Drone, Remote sensing, Foundational models, Radiometric calibration, Computer vision

## Abstract

This dataset comprises a curated collection of UAV RGB and multispectral imagery sourced from multiple open-access platforms. It contains the largest set of UAV imagery covering at least four spectral bands (Green, Red, RedEdge, and Near-Infrared) and is available as either orthomosaic or raw sensor data. A systematic calibration process was employed to ensure radiometric consistency across the diverse sensor types (DJI Mavic 3 M, DJI Phantom 4 Multispectral, Parrot Sequoia, MicaSense RedEdge, and MicaSense Altum(PT)). This involved correcting raw digital numbers (DNs) for sensor-specific variables (e.g., black level, vignetting) and normalizing pixel values with internal sunlight sensors. The resultant imagery is provided as UINT8 5-channel multispectral TIFF files and RGB 3-channel JPG files for robust cross-study comparison.

Comprehensive metadata, processing scripts, and calibration details are publicly available in the accompanying repository folder. This dataset offers a valuable resource for researchers and practitioners seeking consistent, high-quality UAV multispectral data to train or fine-tune foundational models in computer vision and remote sensing applications.

Specifications Table

**Table:** 

Subject	Computer Vision and Pattern Recognition/Computers in Earth Sciences
Specific subject area	UAV-based multispectral and RGB imagery of orchards, vineyards, field crops, forested areas and more. With radiometric calibration for remote sensing applications and foundation model pretraining
Type of data	5-channel multispectral TIFF and 3 channel RGB JPG
Data collection	Multispectral and RGB UAV imagery was collected from online databases and consist of DJI Mavic 3 M, DJI Phantom 4 Multispectral, Parrot Sequoia, and MicaSense Altum/RedEdge sensors from open repositories. Missions which captured orthomosaics or raw sensor data with at least four spectral bands (Green, Red, RedEdge, Near-Infrared) were included. Radiometric calibration employed reflectance panels or onboard sunlight sensors, ensuring normalized radiance.
Data source location	Zenodo
Data accessibility	Repository name: msuav500k: Foundational Dataset for Multispectral and RGB UAV Imagery Data identification number: 10.5281/zenodo.16743975 Direct URL to data: https://doi.org/10.5281/zenodo.16743975 The data can be downloaded from the repository and extracted to any location.
Related research article	Doornbos, J. and Babur, Ö. (2025) Features from Multispectral Drone Data: Curating, training and distributing Transformers for all, EGU General Assembly 2025, Vienna, Austria, 27 Apr–2 May 2025, EGU25–1534, https://doi.org/10.5194/egusphere-egu25-1534, 2025.

## 1 Value of the Data

• **Provides a standardized UAV RGB + multispectral imagery resource** from DJI, Parrot, and MicaSense.
• **Facilitates large-scale computer vision research** by including 512 × 512 patches optimized for deep learning models, simplifying data ingestion and foundational model training.
• **Serves as a benchmark** for remote sensing studies, integrating diverse environmental conditions and flight altitudes**.**

## 2 Background

Standardized, high-quality multispectral Unmanned Aerial Vehicle (UAV) imagery is essential for advancing Deep Learning (DL) applications in agricultural and environmental remote sensing [1]. Contemporary research faces significant challenges due to the heterogeneous nature of sensor systems commonly employed, including DJI Mavic 3 M, DJI Phantom 4 Multispectral, Parrot Sequoia, and MicaSense Altum/RedEdge platforms [2]. This diversity introduces critical issues including radiometric inconsistency across platforms, varied spectral band configurations, and incomplete or non-standardized metadata that collectively impede robust algorithm development [3] and cross-study comparisons [2,4].

The integration of RGB (red, green, blue) imagery alongside multispectral observations presents additional opportunities for enhanced model performance, as RGB sensors provide complementary spatial information that can augment spectral analysis capabilities [5]. However, underlying interoperability challenges such as different methods of radiometric calibration make it difficult to compare observations and create a stable DL model. By systematically gathering and aligning imagery obtained from diverse sensor systems under a unified framework, this dataset directly addresses these fundamental interoperability challenges while enabling improved model generalization across different UAV platforms and sensor configurations. While several specialized UAV datasets exist for specific agricultural applications (CoFly-WeedDB from [6]; Avocado-AirDB from [7]; FlexiGroBots Blueberry from [8], GohbiSet from [9]), msuav500k addresses the critical interoperability challenge by providing radiometrically consistent observations across diverse sensor platforms and application domains.

This standardized, curated dataset establishes a robust foundation for reproducible analyses, systematic algorithm training, and comprehensive model comparisons across the UAV remote sensing community [10]. This standardization effort is particularly critical for DL applications, which require large, consistent datasets to achieve optimal performance and reliable deployment in operational agricultural and environmental monitoring systems.

## 3 Data Description

**Fig. 1:**
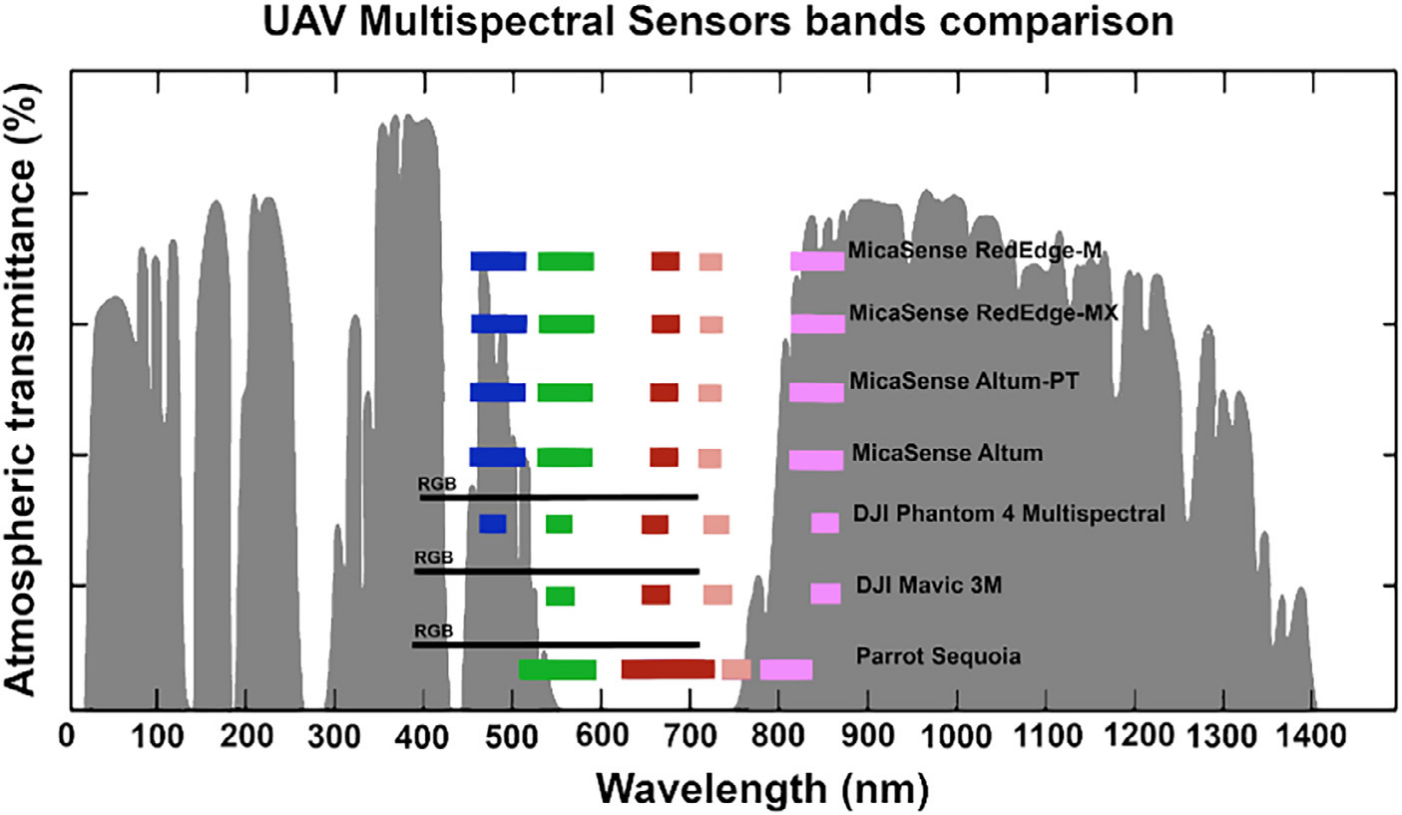
Spectral coverage comparison of multispectral sensors relative to atmospheric transmittance. The grey curve represents atmospheric transmittance across the electromagnetic spectrum. Colored blocks indicate the spectral band coverage (blue, green, red, red-edge, near-infrared) for MicaSense, DJI, and Sequoia multispectral sensors, with block widths corresponding to each sensor's bandwidth. If a sensor contains also an RGB sensor, it is denoted by the black line. X-axis shows wavelength in nanometers.

The msuav500k dataset is built from multiple database searches for RGB an/or multispectral aerial imagery from a UAV. Multispectral aerial imagery in this case is understood as images with spectral information on blue and/or green, red, red-edge and near infrared wavelengths. Whereas RGB imagery is taken with a sensor without the specific wavelength information. In Fig. 1, commonly used multispectral sensors and their wavelengths and widths are visualized over the atmospheric transmittance. The relevant information was acquired from the specifications. Note that some multispectral sensors also contain RGB sensors for additional data. Additionally, the Parrot, and to a lesser extent the MicaSense sensors, use slightly broader bandwidths across all spectra compared to DJI.

The multispectral data is radiometrically calibrated, and the channels spatially aligned and chipped to smaller 512 × 512 images. Chipping is the procedure of taking higher resolution images and cutting it to smaller sub-images. This increases the amount of data, whilst keeping the higher spatial resolution. Multispectral and RGB data is aligned if observations from both were available and chipped to smaller 512 × 512 images. RGB data is kept as-is apart from chipping to smaller 512 × 512 images. This results in a total dataset size of 185GB.

The main folder structure is **MS, RGBMS** and **RGB**. The **MS** folder contains all pre-processed multispectral imagery. Its internal structure is organized by sensor, dataset title, and subset. Within the folder are 5-band calibrated TIFF files (Blue, Green, Red, RedEdge, NIR), named according to the conventions in Table 1. Aligned radiance and RGB data resides in the **RGBMS** folder. These are images which have RGB and multispectral observations from the same flight, with the same calibration procedure as **MS** although with an extra step to ensure spatial alignment between the two data sources. The **RGB** folder contains UAV imagery from RGB sensors. Some examples of the images are visualized in Fig. 1. These images are stored as uint8 data and are min–max scaled with a resolution of 512 × 512. All included datasets are presented in Table 3.

**Table 1:** Filename conventions. E.g. RGB_apple_orchard_01_UAV images_data2018_15m_DJI_0061_1244_1232.JPG and P4M_SUNCAL_p4m_tropical_rain_DJI_0942_138_1056.TIF.

Filename part	Description	Options
SENSOR	Sensor used if multispectral	One of: 3 M, P4M, ALTUM, SEQUIOA, REDEDGE or RGB
CAL	Calibrated or with sun sensor if multispectral	One of: CAL, SUNCAL
TOPIC	Topical title used for internal structuring	e.g. apple_orchard
ORIGINAL-NAME	Original dataset source filename	Original filename
CHIPROWCOL	Integer locating the subset chip in the overall image	Identifier for rows and columns: RRRR_CCCC in pixels.

### 3.1 msuav500k content overview

In total, 598,300 images are contained in the dataset, covering 443,017 RGB observations 136,652 aligned MS and RGB observations (68,326 unique observations from two sensors) and 18,629 MS observations.

### 3.2 RGB set

**Fig. 2:**
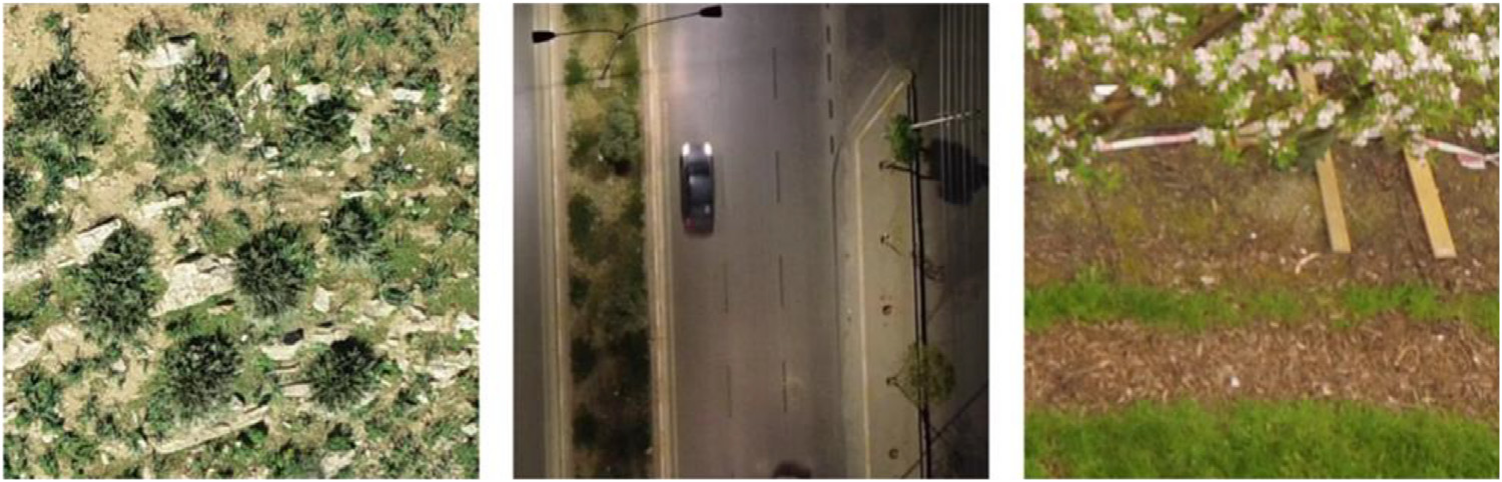
RGB example images after processing. Left: Mavic 3 goats, middle Phantom 4 vehicles, right: Phantom 4 apple orchard.

The **RGB** set comprises 437,067 images collected across 14 distinct projects spanning precision agriculture, infrastructure monitoring, and environmental applications between 2020–2025. For some examples, see Fig. 2. The collection demonstrates standardized sensor deployment with DJI platforms utilized in 93 % of datasets (*n* = 13), including Phantom series (43 %, *n* = 6), Matrice series (21 %, *n* = 3), Mavic series (21 %, *n* = 3), and Zenmuse systems (*n* = 1). One dataset employed MAPIR Survey2 RGB camera (*n* = 1). These are summarized in Table 2.

**Table 2:** RGB Dataset Overview by Content, Purpose, Sensor Type, Ground Sampling Distance, and Image Count. Note: indicates GSD not specified in source documentation.

Content	Purpose	Sensor Type	GSD (cm)	Image Count	Location	Year	Citation
Apple Orchard	Flower Intensity Quantification	DJI Phantom 4 RTK	-	137,900	Netherlands	2022	Zhang et al. [11]
Pear Trees	Rust Disease Detection	DJI Phantom 4 Pro/Matrice 300	0.07–0.2	746	Germany	2024	Maß et al. [12]
Cotton Field	Weed Detection & Species ID	DJI Phantom 4 Pro	-	732	Greece	2022	Krestenitis et al. [6]
Cabbage Field	Crop Detection During Growing	DJI Matrice 300 RTK	-	458	Japan	2024	Yokoyama et al. [13]
Soybean Field	Disease/Pest Detection	DJI Mini 4 Pro	-	79,576	India	2024	Shinde & Attar [14]
Olive Grove	Tree Crown Detection/Segmentation	DJI Phantom 4 RTK	-	7369	Morocco	2025	Hnida et al. [15]
Road Pavement	Distress Detection	DJI Zenmuse H20	-	36,600	China	2023	Yan & Zhang [16]
Urban Roads	Vehicle Detection/Classification	Mavic Air 2	-	6370	Iraq	2024	Mustafa & Alizadeh [17]
Avocado Grove	Fruit Detection/Segmentation	DJI Phantom 4 Pro	2.7	61,992	Morocco	2022	EL Amraoui et al. [7]
Coastal Estuary	Habitat Mapping	MAPIR Survey2 RGB	-	16,956	Mexico	2020	Encinas Lara & Mendez Barroso [18]
Mixed Terrain	Thermal-RGB Sensor Fusion	Zenmuse X3	-	196	Colombia	2020	García-Moreno et al. [19]
Mining Site	Career Segmentation/Object Detection	DJI Phantom 4 RTK	-	40,110	Morocco	2024	Haqiq et al. [20]
Pastoral Land	Livestock Counting	DJI Mavic 3 Enterprise	-	48,062	France	2024	Lebretron et al. [21]

Ground sampling distances (GSDs) exhibited systematic variation by application requirements and operational constraints. Ultra-high resolution applications achieved sub-1 cm GSDs: pear rust detection (0.07–0.2 cm GSD, 746 images using DJI Phantom 4 Pro/Matrice 300 RTK) representing the finest resolution for disease identification. High-resolution applications employed 1–3 cm GSDs: avocado detection (2.7 cm GSD, 61,992 images using DJI Phantom 4 Pro) for fruit counting and segmentation tasks. The majority of datasets did not specify GSD values, indicating focus on relative analysis rather than absolute geometric accuracy. Environmental and infrastructure monitoring applications that reported GSDs utilized moderate resolutions suitable for broad area coverage and object detection rather than fine-scale analysis.

Subject-specific imaging strategies revealed distinct patterns across application domains. Precision agriculture dominated the collection with 7 datasets totalling 288,373 images, where fruit crop monitoring generated the largest volumes: apple orchards contributed 137,900 images, Indian soybean fields 79,576, and avocado groves 61,992. In contrast, disease detection studies employed targeted high-resolution approaches with substantially smaller datasets, as seen in pear rust (746 images) and cotton weed identification (732 images). Infrastructure monitoring encompassed 2 datasets with 42,970 images, spanning pavement distress assessment (36,600) and vehicle classification (6370). An emerging application area, livestock monitoring, demonstrated innovative multi-altitude strategies (30–100 m flight heights) for goat counting across 48,062 images.

### 3.3 RGBMS set

**Fig. 3:**
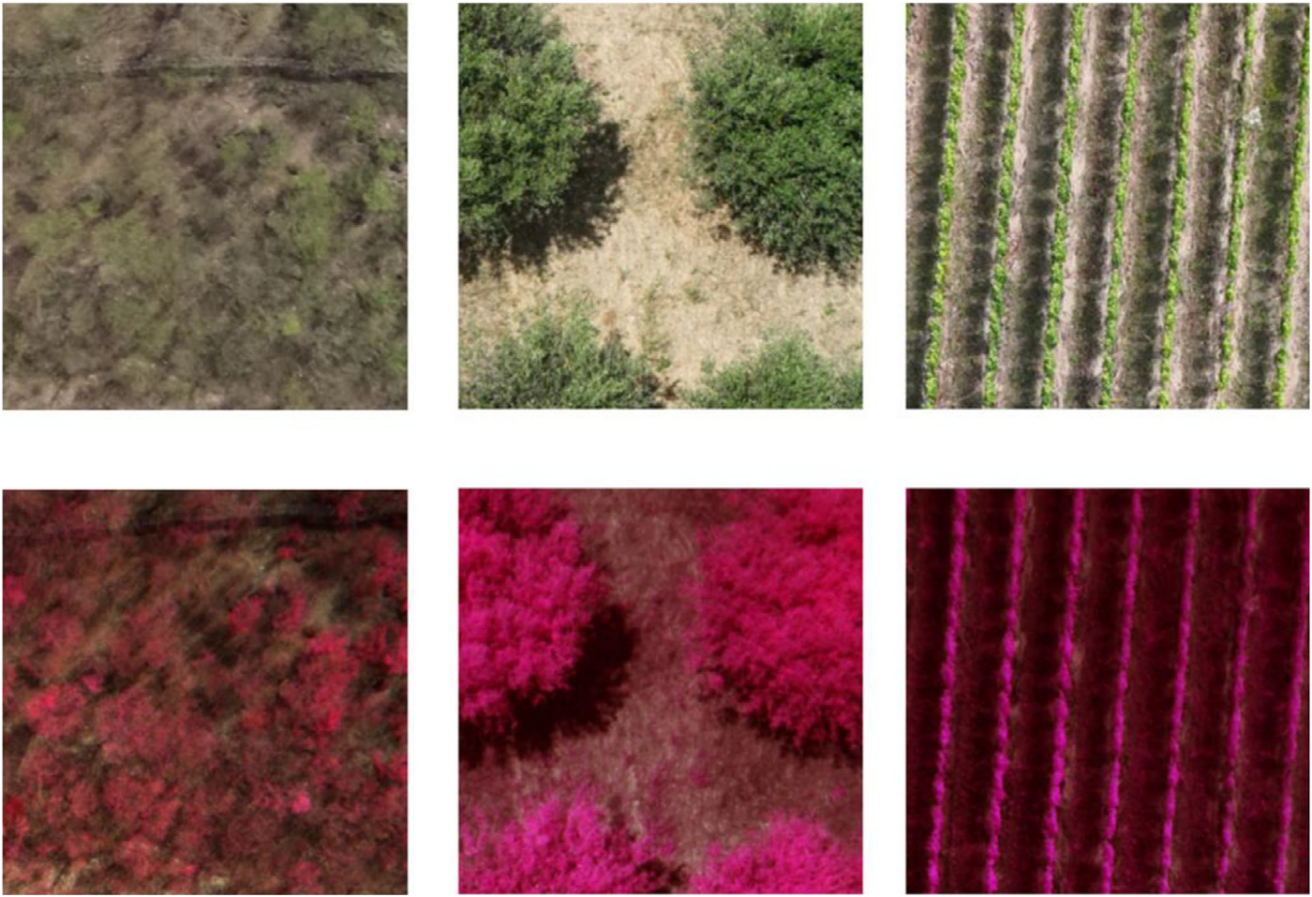
Multispectral false colour + RGB example images after processing. Left: P4M tropical dry forest; middle: 3 M olive grove; right: 3 M mining waste.

The **RGBMS** set comprises 143,590 images collected across 7 distinct agricultural and environmental monitoring projects between 2022–2024. For some examples, see Fig. 3. The collection demonstrates standardized sensor deployment with DJI Mavic 3 M platforms utilized in 70 % of datasets (*n* = 5) and DJI Phantom 4 Multispectral systems in the remaining 30 % (*n* = 2), summarized in Table 3.

GSDs varied systematically by application requirements. Precision agriculture applications demanding fine-scale feature detection employed ultra-high resolution imaging: olive grove disease detection (1.0 cm GSD, 29,190 images) and Portugal vineyard monitoring (0.15 cm GSD, 17,910 images). Standard agricultural monitoring utilized moderate resolutions with UK vineyard datasets achieving 2.0 cm GSD across 58,650 images collected over multiple temporal acquisitions. Environmental monitoring applications employed coarser resolutions suited for broader area coverage: tropical dry forest succession (4.2 cm GSD, 2352 images) and cocoa agroforestry systems (4.2 cm GSD, 15,264 images). The GSD-altitude relationship demonstrated (as expected) inverse correlation, with disease detection flights at 30 m altitude achieving centimeter-level resolution while ecosystem monitoring at 80 m altitude provided 4.2 cm resolution optimal for landscape-scale analysis.

Subject-specific imaging strategies emerged across application domains. Vineyard monitoring (*n* = 3 datasets, 94,470 total images) consistently employed DJI Mavic 3 M systems with sub-2 cm GSDs for phenological assessment and health monitoring. Agricultural crop applications (*n* = 4 datasets, 57,274 images) utilized variable resolution strategies based on diagnostic requirements, ranging from 0.1–1.0 cm GSD for pathogen detection to moderate resolutions for nutrient assessment. Environmental monitoring applications (*n* = 3 datasets, 15,662 images) employed standardized 2.0–4.2 cm GSDs suitable for ecosystem-scale analysis and change detection.

### 3.4 MS set

**Fig. 4:**
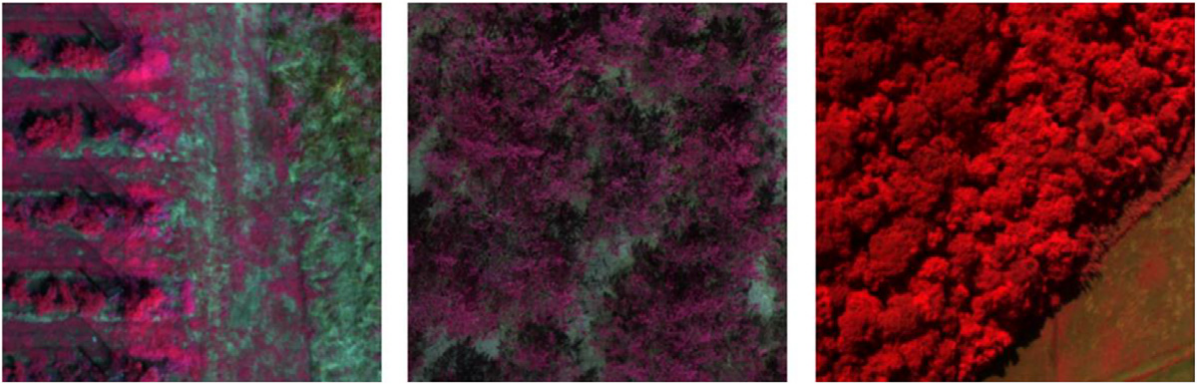
Multispectral false colour example images after processing. Left: RedEdge blueberry; middle: RedEdge contamination (tree section); right: Sequioa tropical forest.

The **MS** set comprises 18,629 images collected across 13 distinct projects spanning agricultural, environmental, and ecological monitoring applications between 2017–2022. For some examples, see Fig. 4. The collection demonstrates sensor deployment with MicaSense platforms utilized in 62 % of datasets (*n* = 8), Parrot Sequoia systems in 38 % (*n* = 5), summarized in Table 4.

Ground sampling distances (GSDs) exhibited systematic variation by application domain and sensor capability. Ultra-high resolution applications achieved sub-2 cm GSDs: Botrytis vineyard disease detection (1.0 cm GSD, 6532 images using MicaSense RedEdge-3), macroalgae monitoring (1.5 cm GSD, 600 images using MicaSense Altum), and beech forest analysis (3.0 cm GSD, 325 images using MicaSense Altum-PT). Precision agriculture monitoring employed moderate resolutions: contamination assessment (5.0 cm GSD, 1924 images) and diurnal crop variability studies (7.5 cm GSD, 61 images). Environmental monitoring applications utilized coarser resolutions optimized for landscape-scale coverage: river habitat mapping (25.0 cm GSD, 95 images using MicaSense RedEdge-M across multiple sites). The GSD-altitude relationship demonstrated expected scaling, with disease detection flights at 30–35 m altitude achieving centimeter-level resolution while broad ecological surveys at 150 m altitude provided 5–25 cm resolution suitable for ecosystem-scale analysis.

Subject-specific imaging strategies revealed distinct patterns across application domains. Disease detection applications (*n* = 4 datasets, 7915 total images) consistently employed high-resolution imaging (1.0–5.0 cm GSD) using primarily MicaSense sensors for pathogen identification in vineyard, cherry, and blueberry crops. Environmental monitoring applications (*n* = 8 datasets, 13,467 images) utilized variable resolution strategies based on scale requirements, ranging from 3.0 cm GSD for forest canopy analysis to 25.0 cm GSD for river habitat classification. Specialized applications including contamination assessment, localization studies, and macroalgae monitoring (*n* = 8 datasets, 12,781 images) employed targeted resolution strategies optimized for specific detection requirements, with GSDs ranging from 1.5–7.5 cm depending on target feature size and analytical objectives.

## 4 Experimental Design, Materials and Methods

### 4.1 Dataset search for multispectral imagery

To systematically identify available UAV multispectral datasets and associated challenges, we adapted the snowball methodology from Johann et al. [40]. We first reviewed papers that addressed UAV-based remote sensing and multispectral image analysis. This enabled us to identify several key sources for datasets, including Zenodo, a widely used open-access repository for research data, and Kaggle, a platform that hosts data science competitions and datasets. Additionally, we utilized targeted searches to identify other relevant repositories and collections that featured datasets related to UAV multispectral imaging applications. Through this process, we discovered Data in Brief, a journal dedicated to data articles, and the ICAERUS Drone Data Analytics Library, a specialized repository for drone-related datasets. Our systematic search strategy employed the combined keywords ``UAV'' + "Multispectral" across all identified platforms to ensure comprehensive coverage and relevance to our research objectives.

On Zenodo, the search was restricted to open-access datasets by setting the filter to ``type = dataset'' This resulted in 849 records. On the *Data in Brief* platform, the same keywords returned 74 records, while Kaggle produced only 2 relevant results.

The datasets were then refined to meet specific criteria, focusing on those that contained at least four spectral bands: Green, Red, RedEdge, and NIR. Additionally, datasets needed to provide imagery in the form of orthomosaics or raw sensor data in the form of imagery. After applying these filters, the search yielded 4 datasets from *Data in Brief*, 23 from Zenodo, and one was identified from ICAERUS library. This resulted in 427GB of extracted files. Some of these datasets contained RGB and multispectral images, some exclusively multispectral images

**Fig. 5:**
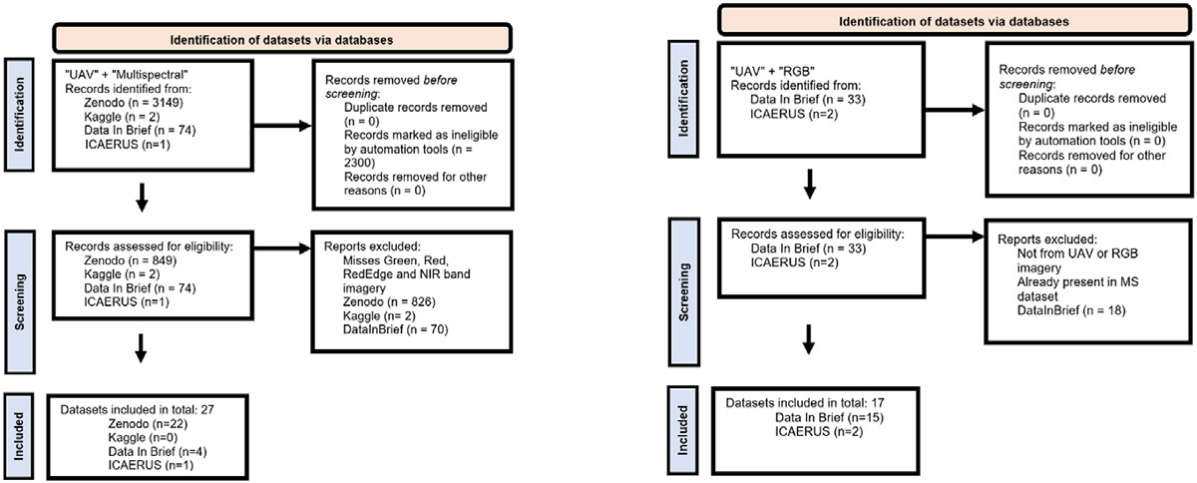
PRISMA inclusion diagrams for multispectral and RGB datasets.

An overview of the selection procedure, including details of the filtering process, is illustrated in Fig. 5, which is based on an adapted PRISMA flow diagram. Furthermore, a summary of the included datasets, along with key metadata, is presented in Table 1, Table 2, Table 3. Full metadata for each dataset can be accessed at the original hosted files.

**Table 3:** RGBMS data overview by Content, Purpose, sensor type, image count and dataset ID. Note: indicates GSD not specified in source documentation.

Content	Purpose	Sensor Type	GSD (cm)	Image Count	Location	Year	Citation
Olive Grove	Disease Detection	DJI Mavic 3M	1.0	29,190	Italy	2024	Taal S.r.l [22]
Vineyard	Multi-temporal Monitoring	DJI Mavic 3M	2.0	58,650	UK	2024	Crane [[23], [41], [42], [43], [44]]
Vineyard	Precision Viticulture	DJI Mavic 3M	0.15	17,910	Portugal	2024	Marengo & Faria [24]
Mining Waste Site	Environmental Assessment	DJI Mavic 3M	2.0	10,858	Germany	2023	Ablanedo Enoc Sanz [45]
Rice Paddy	Nitrogen Requirements Analysis	DJI Mavic 3M	0.1	2430	Sri Lanka	2023	Fonseka et al. [25]
Tropical Dry Forest	Succession Monitoring	DJI Phantom 4M	4.2	2352	Mexico	2022	Vega-Puga et al. [26]
Cocoa Agroforestry	Typology Classification	DJI Phantom 4M	4.2	15,264	Côte d'Ivoire	2022	Lammoglia et al. [27]

### 4.2 Dataset searches RGB

A systematic search was conducted on two platforms: Data in Brief and the ICAERUS Drone Data Analytics Library to identify UAV RGB datasets. The search utilized the keywords “UAV” + “RGB” to ensure relevance. Due to the number of high quality datasets from this database, and to keep the multispectral to RGB imagery balanced, one search was all that was required.

On the *Data in Brief* platform, the keyword search returned 33 records. The datasets were then refined to meet specific criteria, focusing on those that contained RGB image files from UAV observations. Additionally, datasets needed to provide imagery in the form of orthomosaics or raw sensor data in the form of imagery. After applying these filters, the search yielded 15 datasets from *Data in Brief*, and two from the ICAERUS library. This resulted in 60 GB of extracted files.

An overview of the selection procedure, including details of the filtering process, is illustrated in Fig. 5, which is based on an adapted PRISMA flow diagram. Furthermore, a summary of the included datasets, along with key metadata, is presented in Table 2, Table 3, Table 4. Full metadata for each dataset can be accessed at the original hosted files.

**Table 4:** Extended MS Dataset Overview by Content, Purpose, Sensor Type, Ground Sampling Distance, and Image Count. Note: indicates GSD not specified in source documentation.

Content	Purpose	Sensor Type	GSD (cm)	Image Count	Location	Year	Citation
Vineyard	Botrytis Disease Detection	MicaSense RedEdge-3	1.0	6532	Spain	2021	Vélez et al. [28]
Crop Fields	Diurnal Variability Study	Parrot Sequoia + Mixed	7.5	61	Netherlands	2017	Kallimani et al. [29]
Urban Area	UAV Localization	Parrot Sequoia	-	448	Brazil	2019	Westhauser [30]
Beech Forest	Forest Monitoring	MicaSense Altum-PT	3.0	325	Germany	2022	Jackisch [31]
Cherry Orchard	Disease Detection	Parrot Sequoia	-	16	Greece	2022	Chaschatzis et al. [32]
Blueberry Orchard	Disease Detection	MicaSense RedEdge-M	-	343	Serbia	2021	Bojana et al. [8]
River Systems	Habitat Classification	MicaSense RedEdge-M	25.0	95	Germany	2019	Rock et al. [[33], [46], [47], [48], [49], [50]]
Potato Field	Crop Monitoring	MicaSense RedEdge-MX	-	347	Slovenia	2022	Lapajne et al. [34]
Subtropical Forest	Forest Monitoring	Parrot Sequoia	-	1490	Brazil	2020	Breunig et al. [35]
Nature Reserve	Biodiversity Assessment	Parrot Sequoia	-	2009	Belgium	2017	Vanden Borre et al. [36]
Forest Canopy	Fuel Parameter Mapping	MicaSense RedEdge-MX	-	4439	USA	2019	Reilly et al. [37]
Intertidal Zone	Macroalgae Classification	MicaSense Altum	1.5	600	Spain	2019	Martinez-Movilla et al. [38]
Contaminated Site	Post-remediation Evaluation	MicaSense RedEdge-M	5.0	1924	Poland	2022	Kondracka [39]

### 4.3 Calibration of multispectral data

**Fig. 6:**
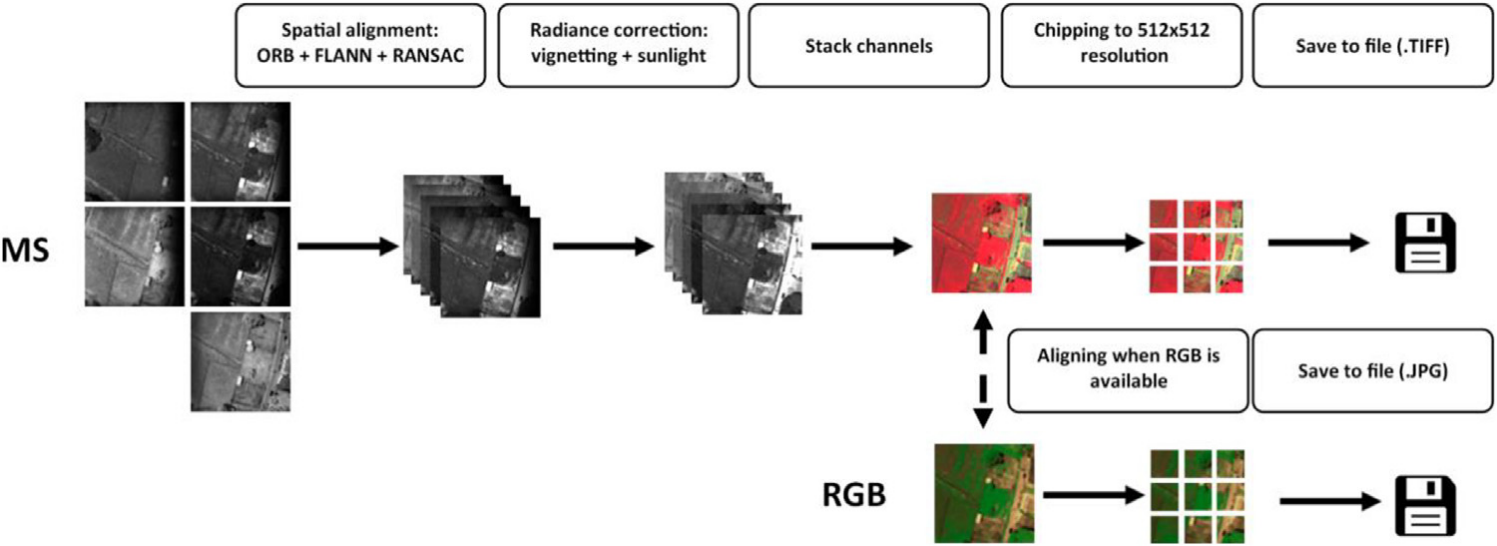
Image processing pipeline. Raw multispectral observations are taken and spatially aligned using ORB, FLANN and RANSAC methods. Digital Numbers are converted to radiance using vignette correction and sunlight information from the sensor metadata. If RGB images are available of that observation, they are also aligned to the same spatial location. Images are then chipped to a 512 × 512 resolution. For the RGB pipeline, only the chipping is performed. Finally, all chips are saved to disk.

Multispectral sensors capture digital numbers during data acquisition and are acquired using separate imaging sensors. These two factors therefore require multispectral bands to be spatially aligned and spectrally corrected to standardize observations from the different datasets. RGB on the other hand requires very little preprocessing. The complete preprocessing pipeline is presented in Fig. 6.

### 4.4 Spatial alignment

When capturing multispectral images, each spectral band may experience slight spatial offsets due to sensor misalignment or movement during flight. To ensure consistency across bands, an image alignment process is applied. This involves detecting distinctive features within the images using the ORB (Oriented FAST and Rotated BRIEF) algorithm to extract keypoints and descriptors. These features are then matched between images using a FLANN-matcher to identify corresponding points.

Once good matches are found, an affine transformation is calculated using RANSAC (Random Sample Consensus), which accounts for translation, rotation, scaling, and minor distortions. The resulting transformation matrix is applied to align one band to another, ensuring that each pixel represents the same ground location across all bands. These steps are visualized as Spatial alignment in Fig. 6.

### 4.5 Radiance correction

After alignment, the next essential step is radiometric calibration, which converts raw pixel values into accurate reflectance values. The raw sensor readings are influenced by factors such as black level, sensor gain, exposure time, vignetting effects, and irradiance. These factors are corrected through a series of processing steps to ensure the data accurately reflects radiance.

First, raw pixel values are normalized by subtracting the sensor's black level and adjusting for gain and exposure time. Next, vignetting correction is applied to address brightness variations across the image frame, using metadata-derived coefficients to model the radial falloff in brightness from the image center.

Finally, an irradiance correction is applied to convert the corrected pixel values into reflectance values. This step adjusts for varying light conditions at the time of capture by dividing the image by the measured irradiance. The resulting reflectance images are radiometrically consistent, enabling reliable comparisons across time, locations, and environmental conditions. These are visualized as radiance correction in Fig. 6.

### 4.6 Stacking and chipping

The multispectral imagery is now in a similar state to the RGB imagery. With another alignment required between MS and RGB imagery, for when the dataset contains both. This is performed in the same way as the Spatial alignment above. All imagery is then ready for chipping, where the larger resolution imagery is cut into 512 × 512 pixel images.

The difference between extracted file size (461GB) and processed output file size (185GB) has to do with more efficient saving: the use of uint8 instead of uint16 from the original multispectral images. The conversion from uint16 to uint8 reduces storage requirements by 60 % while maintaining dynamic range for deep learning applications. Radiometrically calibrated reflectance values are min-max scaled to fully utilize the uint8 range, preserving meaningful spectral distinctions for agricultural and environmental monitoring tasks.

### 4.7 MicaSense calibration differences

The process for the MicaSense RedEdge, was identical to the DJI processes, the only variation being an adjustment for the EXIF information and type of distortion correction (pinhole). The datasets captured with the MicaSense Altum (PT) sensor already include pre-calibrated reflectance values.

### 4.8 Parrot Sequoia calibration differences

For the raw Sequioa data, a similar approach was taken to the previous sensors, with the major difference accounting for a fisheye lens distortion.

## Limitations

Despite the large volume, most included datasets are single-season snapshots. There is a heavy European/Mediterranean concentration (40 % of datasets) with minimal representation from tropical, arid, and high-latitude agricultural systems. Also, there is a disproportionate emphasis on high-value perennial crops (vineyards, orchards) versus staple grains (wheat, rice, maize) which poorly reflect the foundation of global food systems but could be interpretated as the first possible use-cases for UAVs in practice.

85 % of the multispectral datasets lack external radiometric calibration panels, limiting reflectance calculation and inter-comparison on reflectance levels, causing this dataset to focus on radiance calculation instead.

Furthermore, DJI remains the main provider for sensors, being the sole constitutor for RGBMS aligned imagery.

## Ethics Statement

The authors confirm that they have read and adhere to the ethical requirements for publication in Data in Brief. They further confirm that the present work does not involve human subjects, animal experiments, or any data derived from social media platforms.

## CRediT Author Statement

**JD:** Data Curation, Literature Search, Writing – Original Draft; **OB:** Writing - Review, Supervision, Project Administration.

## Acknowledgements

This work has been carried out in the scope of the HORIZON ICAERUS project, which has been funded by the European Commission in the scope of its HORIZON program (contract number 101,060,643, https://icaerus.eu/). The authors acknowledge valuable help and contributions from all partners of the project.

## Data Availability

Zenodomsuav500k (Original data).
